# Intelligent assistant diagnosis for pediatric inguinal hernia based on a multilayer and unbalanced classification model

**DOI:** 10.3389/fphys.2023.1105891

**Published:** 2023-03-14

**Authors:** Zhi-Wen Liu, Gang Chen, Chao-Fan Dong, Wang-Ren Qiu, Shou-Hua Zhang

**Affiliations:** ^1^ Department of General Surgery, Jiangxi Provincial Children’s Hospital, Nanchang, China; ^2^ Computer Department, Jing-De-Zhen Jingdezhen Ceramic Institute, Jingdezhen, China; ^3^ Department of General Surgery, Jingdezhen No. 1 People’s Hospital, Jingdezhen, China

**Keywords:** imbalanced data, medical numerical data, postoperative diagnosis, machine learning, intelligent assistant diagnosis

## Abstract

As one of the most common diseases in pediatric surgery, an inguinal hernia is usually diagnosed by medical experts based on clinical data collected from magnetic resonance imaging (MRI), computed tomography (CT), or B-ultrasound. The parameters of blood routine examination, such as white blood cell count and platelet count, are often used as diagnostic indicators of intestinal necrosis. Based on the medical numerical data on blood routine examination parameters and liver and kidney function parameters, this paper used machine learning algorithm to assist the diagnosis of intestinal necrosis in children with inguinal hernia before operation. In the work, we used clinical data consisting of 3,807 children with inguinal hernia symptoms and 170 children with intestinal necrosis and perforation caused by the disease. Three different models were constructed according to the blood routine examination and liver and kidney function. Some missing values were replaced by using the RIN-3M (median, mean, or mode region random interpolation) method according to the actual necessity, and the ensemble learning based on the voting principle was used to deal with the imbalanced datasets. The model trained after feature selection yielded satisfactory results with an accuracy of 86.43%, sensitivity of 84.34%, specificity of 96.89%, and AUC value of 0.91. Therefore, the proposed methods may be a potential idea for auxiliary diagnosis of inguinal hernia in children.

## 1 Introduction

The incidence of inguinal hernia in children is common. Galinier et al. (2007) pointed out that the incidence of inguinal hernia in children of any age is about 0.8%–4.4%, and in premature babies, it is even as high as 30%. Generally, inguinal hernia in pediatric patients is caused by their congenital abnormalities. Although some new methods are studied in this issue (Chowdhury et al., 2019; Molinaro et al., 2022; Zhao et al., 2022), for patients with different conditions, treatment methods also differ. If there is only hernia and no serious diseases such as intestinal necrosis, conservative treatment will be adopted. If serious diseases such as incarcerated necrosis of the intestines occur, surgical treatment will be adopted to prevent the risk of internal damage to the renal organs of pediatric patients. Usually, the diagnosis of intestinal necrosis of inguinal hernia is determined by medical imaging equipment, doctor’s clinical experience, or symptoms after surgery. Because medical imaging examinations have a greater radiation impact on children than on adults, m any parents disagree with children’s medical imaging examinations. At this time, medical expertise is very important for the diagnosis of intestinal necrosis in pediatric patients, and it is also a test for experts.

With the continuous advancement of concepts in the area of precision medicine, the application of intelligent algorithms in medical diagnosis has become increasingly extensive. By constructing predictors on clinical data, the purpose of assisting diagnosis is achieved. Common MRI imaging data (Gurses et al., 2019; Wadhwa et al., 2019; Wang et al., 2022), CT imaging data (Mohakud et al., 2019; Masselli et al., 2020; Singh et al., 2020; Zhuang et al., 2021), and EEG imaging data (Prucnal and Polak, 2019; Quintero-Rincón et al., 2020) are helpful in the work. However, there are a few auxiliary diagnosis models based on medical digital and textual data. However, some researchers introduced special cases in more detail or performed simple analysis on the current patient’s condition (Shiqi et al., 2018; János et al., 2020; Sabra et al., 2020; Abdulrahman et al., 2021; Beau et al., 2021; Karhade et al., 2021; Radhakrishnan et al., 2021; Hyun et al., 2022; Lin et al., 2022; Oh et al., 2022). No corresponding auxiliary diagnosis model was constructed based on these data because the data information that can be mined by case analysis or statistical analysis is very limited. Scrutinio et al. (2020) used machine learning algorithms to build a decision-making model for the prognosis of stroke survivors, providing better guidance for doctors in clinical diagnosis. We can combine machine learning algorithms to collect more information from digital and textual clinical data (Ricciardi, 2019; Onan, 2020; Onan and Tocoglu, 2021), such as some important examination parameters or making diagnosis decisions for patients, which is extremely significant to the doctor’s accurate diagnosis. Some doctors had conducted a retrospective bicentric study in this point (Bouassida et al., 2022).

In this work, we used the clinical data of pediatric patients with inguinal hernia and non-inguinal hernia. However, clinical data are different from other data. From the actual examination items performed by the patient to the collection of clinical data, some vacancies can easily occur in the examination parameters. In order to better collect more information from limited data and build a corresponding model, the nature of the data needs to be followed in the research process. If researchers blindly pursue the complexity and diversity of sample parameters, the possible consequence is that there are too few samples that can be used in the experiment, which is not conducive to experimental research. Selecting appropriate characteristic values from some common examinations of patients is a data mining method worth exploring. Therefore, in this study, we defined a model using blood routine test parameters as M1, a model using liver and kidney function test parameters as M2, and a model using blood routine test parameters and liver and kidney function test parameters as M3.

We first used statistical analysis methods to preprocess the original data and used the RIN-3M (median, mean, and mode region random interpolation) method to fill in the vacancy in the data. Second, the importance of features was compared according to the Gini coefficient (Fang et al., 2012), and the combination of features with the best performance was selected in an iterative manner. Third, we used an ensemble learning method (Onan, 2017) to deal with the problem of sample imbalance. Finally, the samples after feature selection and the original samples were put into the RF algorithm to train them as predictors. Comparing the performance of each model, we found that the model after feature selection had better performance.

**FIGURE 1:**
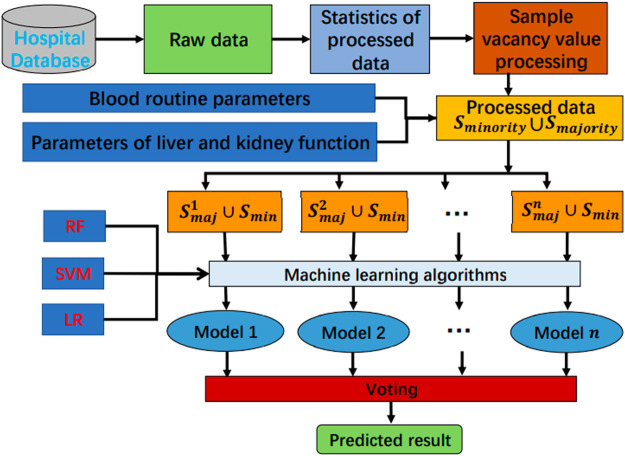
Flow chart of data analysis.

The analysis process of these data is shown in Figure 1, where $S_{majority}$ and $S_{minority}$ represent the processed majority class sample and minority class sample, respectively; $S_{maj}^{1},S_{maj}^{2},...,S_{maj}^{3}$ represent the sub-samples equally divided in the training set of the samples of the majority class; and $S_{\min}$ represents the samples used for training in the samples of the minority class. The specific profile of most samples divided into sub-samples under different parameters is shown in Table 1.

**TABLE 1:** Profile of positive and negative samples.

	Intestinal necrosis		Incarcerated inguinal hernia	
	Training data	Test data	Training data	Test data
			Sub-sample*number	
M1	97	24	100*19	483
M2	135	34	149*11	415
M3	130	33	130*5	168

## 2 Materials and methods

### 2.1 Logistic regression (LR)

The logistic regression algorithm (Abu-Hanna and de Keizer, 2003; Zhu and Fang, 2016; Li et al., 2019) plays an important role in aiding decision-making in clinical medicine, where researchers construct linear regression functions using clinical examination parameters as characteristic input parameters and map the values obtained from the linear regression functions between 0 and 1 by means of a sigmoid function, thus achieving classification. It is commonly used to construct equations for the relationship between input vectors and categories. The principle of this study is shown in Eqs 1, 2.

$$\begin{array}{c} y=c_{0}+c_{1}x_{1}+c_{2}x_{2}+c_{3}x_{3}+\ldots +c_{n}x_{n} \end{array},$$ (1)

$$\begin{array}{c} L=sigmoid\left(y\right)=\frac{1}{1+e^{-y}} \end{array}.$$ (2)

In formula (1), $c_{0},c_{1},\ldots ,c_{n}$ are the parameters of the polynomial fitting curve, $\left(x_{1},x_{2},\ldots ,x_{n}\right)$ represent the n-dimensional input eigenvectors, and y represents the output value of the fitting equation. The output value L of the sigmoid function is 1 when y is greater than 0; otherwise, it is 0.

### 2.2 Support vector machine (SVM)

When dealing with classification problems, SVM maps samples to higher dimensions if they are indistinguishable in the current dimension, so that the samples are linearly separable in the higher dimensional space. Also, a segmentation hyperplane is constructed in the samples in the high-dimensional space to maximize the distance between the sample points and the hyperplane for the purpose of classification. Because of its good learning ability, the SVM algorithm is widely used in clinical disease diagnosis, and the algorithm has strong processing performance in the face of complex clinical medical data (Zhu et al., 2013; Recenti et al., 2019; Reynolds et al., 2019; Chen and Lin, 2020).

### 2.3 Random forest (RF)

Random forest (Zhao et al., 2020) is itself a swarm policy algorithm. It constructs optimal decision trees by releasably drawing n samples at random from the sample and constructing the optimal decision tree for each drawn dataset. Many optimal decision trees are combined to form a random forest. Due to the relatively stable performance of the models constructed by the RF algorithm, many researchers often apply such algorithm to disease analysis (Asadi et al., 2021; Quist et al., 2021).

## 3 Experiment design

### 3.1 Datasets

The data of this study were derived from the diagnostic data on children with incarcerated inguinal hernia in Jiangxi Children’s Hospital. The study was approved by the Ethics Committee of Jiangxi Children’s Hospital with ethics approval number JXSETYY-YXKY-20210016. Because the subjects were all under 18 years of age, informed consent from their guardian or legal close relatives was obtained. In order to protect the patients’ private information, we used digital codes to replace the names and other private information.

We selected 3,807 children with incarcerated inguinal hernia but no intestinal necrosis as the positive sample set, denoted as S1, and 170 children with incarcerated inguinal hernia caused by intestinal necrosis as the negative sample set, denoted as S2. The clinical parameters used in this study are blood routine examination parameters and liver and kidney function examination parameters, and the patient discharge diagnosis results are the basis for the category label of the study.

However, it is quite often that the dimensions of the examination parameters are inconsistent in the diagnostic data, which may be due to the lack of certain examination items in the hospital. In other words, some children only have a single test item such as that of blood or liver and kidney function. Of course, there are also patients who have multiple test items at the same time. Therefore, based on the characteristics of the clinical data on children with incarcerated inguinal hernia, the blood routine single clinical examination data, the single clinical examination data on liver and kidney function, and the combination of these two examination parameters served for modeling and analysis in this work.

### 3.2 Data preprocessing

#### 3.2.1 Statistical magnitude

The original data have been analyzed with statistical theory. Based on the number of features and samples, some clinical examination parameters with low sample size were excluded. The clinical examination parameters after preliminary screening are shown in Table 2

**TABLE 2:** Clinical parameters.

Inspection method	Parameter name
Blood routine examination (f1–f20)	Basophils, basophil ratio, eosinophils, eosinophil ratio, hematocrit, hemoglobin, large platelet ratio, lymphocyte count, lymphocyte ratio, mean corpuscular hemoglobin, mean corpuscular hemoglobin concentration, mean corpuscular volume, mean platelet volume, monocyte count, monocyte ratio, neutrophil count, neutrophil ratio, platelet, platelet crit, and platelet distribution width
Liver and kidney function (f21–f50)	Alanine aminotransferase, albumin, albumin to globulin ratio, alkaline phosphatase, aspartate aminotransferase, calcium, chloride, creatine isoenzyme, creatine kinase, C-reactive protein, creatinine, direct bilirubin, globulin, glutamyl transpeptidase, indirect bilirubin, lactate dehydrogenase, myoglobin, potassium, prealbumin, retinol-binding protein, sodium, total bilirubin, troponin I, total protein, transaminase ratio, urea, uric acid, urea: creatinine, 5′-nucleotidase, and β2 microglobulin
Others (f0)	Age

Unfortunately, there were still some null values in the parameters of a certain examination because the patient did not undergo a certain examination or there are some deviations in the information input or information collection. For those samples with missing values, the usual processing method used is deletion or interpolation. Because there is an imbalance between positive and negative samples, it may be more severely imbalanced when some samples with null or missing values of the minority are deleted. Thus, in this work, the selection of samples depends on the missing rate of the inspection parameters in the samples. The selected sample should be the positive sample without any missing value or the negative sample with less than 40% of the whole features missing. In this way, some sparse feature samples can be eliminated, and the integrity of negative sample information can be preserved to a large extent to avoid further expansion of sample imbalance.

#### 3.2.2 Data interpolation

For those samples with missing values, the commonly used processing methods are interpolation (Liu et al., 1997; Rababah Msc et al., 2019), mean, mode, and median, and nearest neighbor imputation (Beretta and Santaniello, 2016). Although nearest neighbor imputation has good performance in image processing, there are some limitations to this experiment because of the number of samples. If the mode interpolation is used, because the number of negative samples is only 170, the mode of some selected features may not be representative. Second, because the clinical examination parameters are discrete, some features may have multiple modes.

Therefore, choosing an appropriate mode is also a difficult task. In this study, we combined the actual situation of the data, in order to make the interpolation closer to reality, comprehensively considered the different characteristics of the mode, mean, and median, and adopted a new interpolation method for these vacant data. This is the regional random interpolation (RIN-3M) method of the median, mode, and mean. The principle of this method is shown in formula (3).

$$I_{i}=\left(\max\left(m^{\left(i\right)},n^{\left(i\right)},p^{\left(i\right)}\right)-\min\left(m^{\left(i\right)},n^{\left(i\right)},p^{\left(i\right)}\right)\right)*r_{v}+\min\left(m^{\left(i\right)},n^{\left(i\right)},p^{\left(i\right)}\right).$$ (3)

In formula (3), $I_{i}$ is the value which needs to be inserted for the ith feature; $m^{\left(i\right)}$ , $n^{\left(i\right)}$ , and $p^{\left(i\right)}$ are the mode, mean, and median of the ith feature, respectively; and $r_{v}$ is a random number between 0 and 1.

### 3.3 Feature selection

**FIGURE 2:**
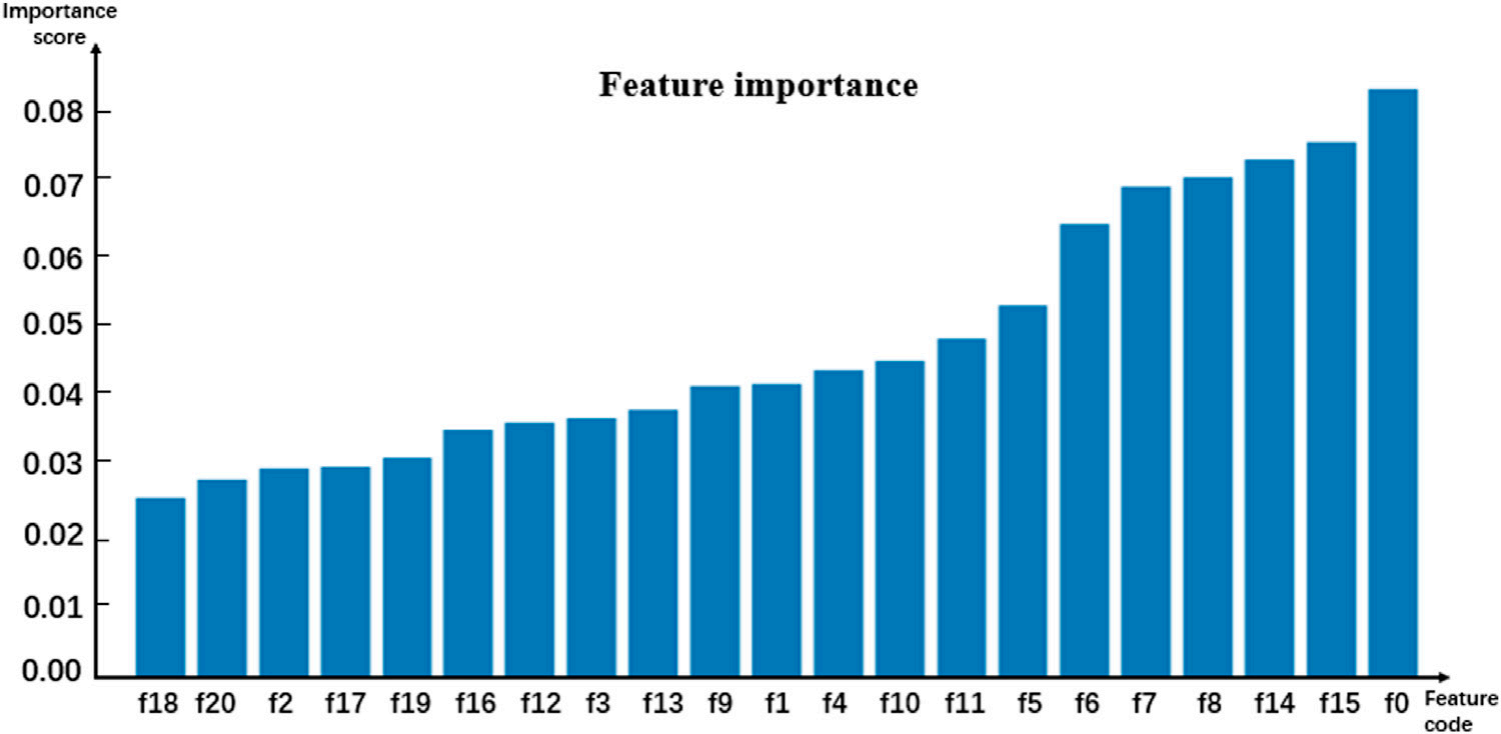
Histogram showing the importance of parameters in model M1.

**FIGURE 3:**
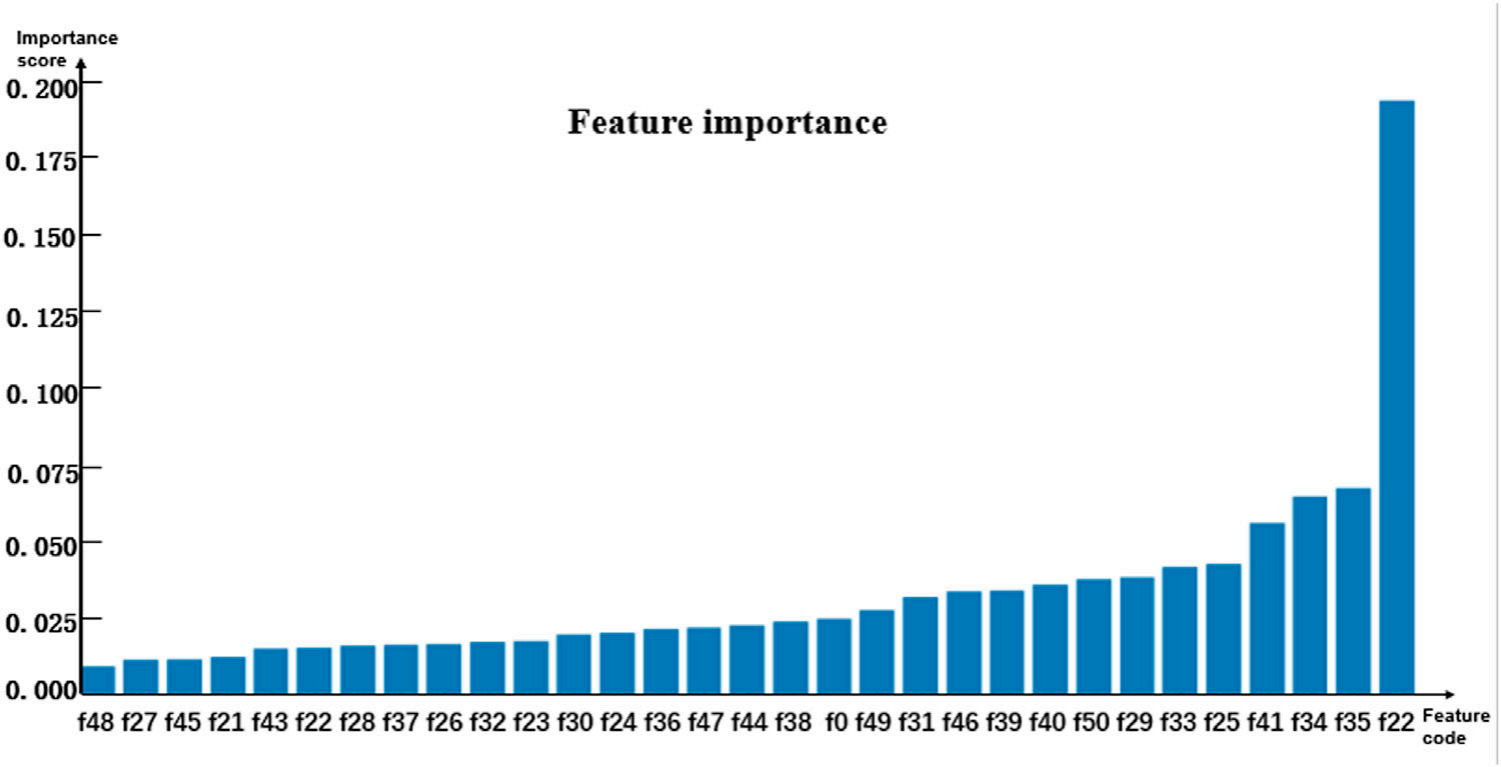
Histogram showing the importance of parameters in model M2.

**FIGURE 4:**
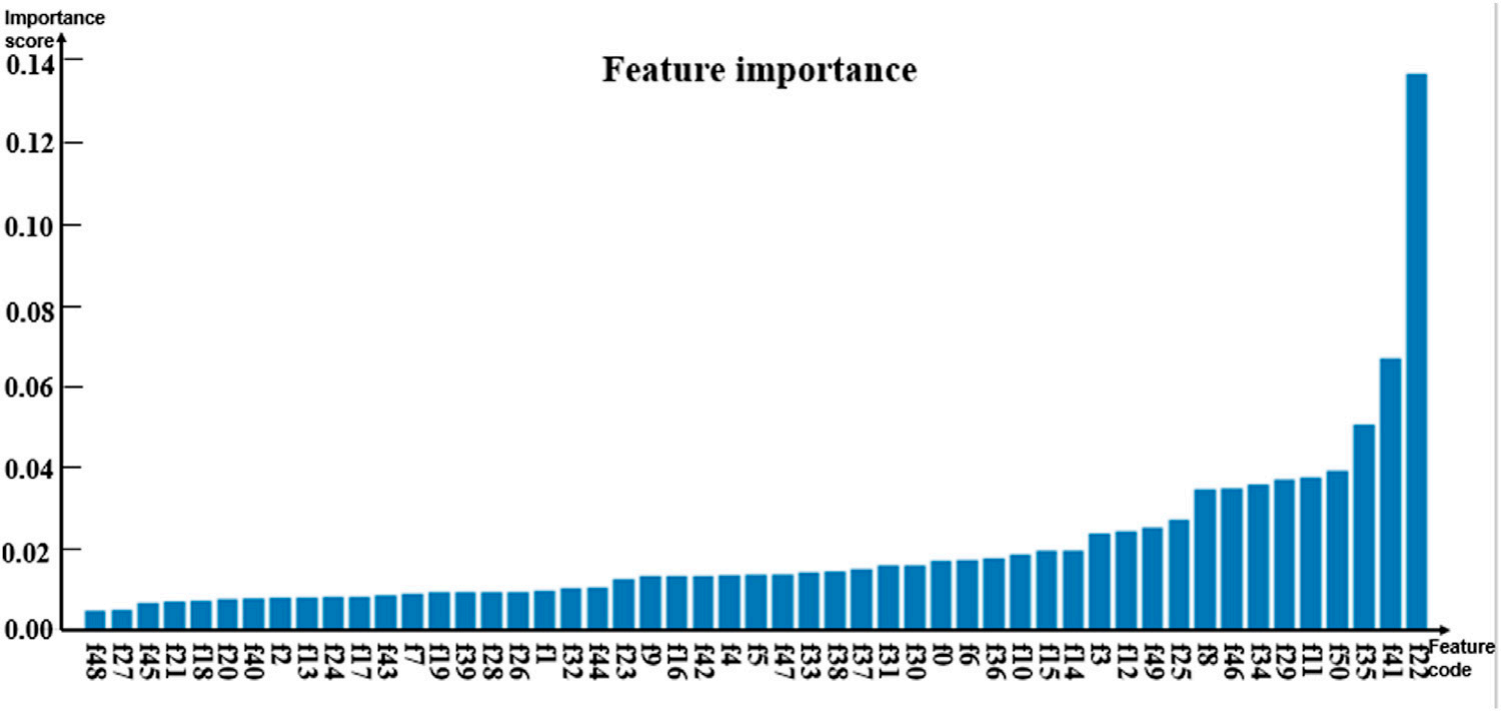
Histogram showing the importance of parameters in model M3.

Random forests have relatively stable performance at the time of dealing with heterogenetic parameters because the constructed decision trees could randomly extract some feature values and avoid the influence of too many redundant features in the process of training models (Onan et al., 2016a; Onam and Serdar, 2016; Onan, 2016; Onan and Korukoğlu, 2016; Onan, 2019; Toolu and Onan, 2021). Thus, the random forest algorithm classifier was selected as a sub-model. To analyze the contribution of the involved parameters to different models, the importance of features was evaluated by using the feature_importance method based on the Gini coefficient theory in the sklearn library of Python. The importance score of each feature was obtained by calculating the sum of the degree of impurity reduction of each feature, and then the importance of the parameter depends on the importance of the sub-model. The average of five-fold cross-validation test results served as the importance score of the model. The histograms in Figures 2–4 show the importance of parameters in the M1, M2, and M3 models.

Figure 2 shows that the age marked as f0 is the most important feature. In Figures 3, 4, the C-reactive protein labeled f22 is the most important parameter. Since the level of C-reactive protein reflects the degree of infection, it is an important indicator of certain diseases. In fact, the feature importance histograms of the models M2 and M3 also fully indicated that the feature importance of C-reactive protein is relatively high, so C-reactive protein can be used as a reference for the diagnosis of inguinal hernia. Second, the feature importance of direct bilirubin, albumin, and troponin I in the second gradient was high, as shown in Figure 3, which are labeled as f35, f34, and f41, respectively. The feature importance of troponin I and direct bilirubin in the second gradient was also high, as shown in Figure 4; it can be seen that these two examination parameters can also provide doctors with reference values.

In order to better compare the features that have significant contributions to these three models, Table 3 lists the top 10 important parameter names in the M1 and M2 models, the top 15 important parameter names in the M3 model, and their importance score. The feature intersection between M1 and M3 in Table 3 and the feature intersection between M2 and M3 are analyzed in the Discussion sub-section.

**TABLE 3:** Characteristic importance ranking of M1, M2, and M3 parameters.

M1		M2		M3	
Feature	Importance	Feature	Importance	Feature	Importance
Age	0.08476063	C-reactive protein	0.19369296	C-reactive protein	0.13330727
Mean corpuscular volume	0.07512136	Direct bilirubin	0.06635172	Troponin I	0.06787233
Hematocrit	0.07433977	Albumin	0.06205365	Direct bilirubin	0.04879934
Monocyte count	0.06902576	Troponin I	0.05554238	Retinol-binding protein	0.03912257
Monocyte ratio	0.06892273	Prealbumin	0.04254516	Basophil ratio	0.03847966
Lymphocyte count	0.06348745	Albumin to globulin ratio	0.0390976	Total bilirubin	0.03825586
Lymphocyte ratio	0.05230277	Total bilirubin	0.03887077	Albumin	0.03810347
Basophil ratio	0.04958729	Retinol binding protein	0.03805697	Calcium	F
Basophils	0.04446896	Creatine isoenzyme	0.03481849	Monocyte count	0.03749601
Neutrophil ratio	0.04412248	Creatine kinase	0.034006	Prealbumin	0.02736976
				Indirect bilirubin	0.0244206
				Basophils	0.02433038
				Neutrophil ratio	0.0238246
				Mean corpuscular volume	0.0209632
				Mean hemoglobin	0.01883659

### 3.4 Classification model design and training

To solve the problem of imbalanced forecasting and improve the prediction accuracy, some researchers have recently tried to use the combination of up-sampling and down-sampling methods (Rubin et al., 2009). In the procedure of balancing the training datasets, some synthetically generated data points are injected into the minority class dataset in the up-sampling method, while the down-sampling method would train on a disproportionately low sub-set of the majority class examples by adding a weight to the down-sampled class. Both methods have advantages and disadvantages. This study uses a voting-based integrated learning method to solve data imbalance. The model is trained by dividing most samples into multiple sub-sets to improve the training effect of the model.

In this study, 20% of the positive and negative samples are selected as test sets, and the remaining 80% served as the training sets. Subsequently, the positive sample set with majority categories was divided into multiple sub-sets, and the numbers of samples in all sub-sets are almost equal to the number of negative samples. Every dataset of the sub-sets and the negative set form a sub-balance training dataset, denoted as $S_{maj}^{i}\cup S_{\min}$ , for training the ith sub-model. Then, the ensemble classifier (Onan et al., 2016b; Onan et al., 2017; Onan and Mansur, 2020) is formed by fusing the sub-models trained by the sub-balance dataset, and the final prediction results would serve for doctors’ decision-making.

As shown in Table 2, the parameters of this research issue comprise blood routine examination and liver and kidney function; the classification models should be trained according to the parameters. Actually, the model should be separately trained with the blood routine examination parameters and the liver and kidney function parameters because the dimensionality of the data is very heterogenetic and it is not appropriate or practical for diagnosis. The inspection is often based on the process. Some basic examinations should be performed on the patient, and a more in-depth examination will be performed when a doctor makes a diagnosis. As basic checks, some features of routine blood tests, for example, white blood cell count and red blood cell ratio, are usually used as diagnostic reference indicators for doctors. However, liver and kidney function tests are relatively stricter than routine blood tests. The liver and kidney function tests are different from the routine blood test because the patient’s venous blood needs to be taken for testing and the patient also needs to fast before the blood is drawn. However, compared with B-ultrasound and other medical imaging diagnostic methods, the results of blood routine and liver and kidney function tests are superior to the former in terms of economy and operation process. Then, a specific model based on parameters would reduce the medical resources and patients’ financial expenditure. Moreover, in many hospitals, the parameters of routine blood tests and liver and kidney function tests are easily available since the tests are cheap and convenient.

To make the auxiliary diagnosis models in line with the actual situation of the diagnosis process and simplify the complexity, the proposed models are trained with different inhomogeneous features. Based on the aforementioned data explanation, the blood routine parameters are used to train the first kind of model named M1, the liver and kidney function parameters served for training the second model denoted as M2, and all of the parameters for the third model marked as M3. Table 1 shows the profiles of the datasets.

## 4 Results and discussion

### 4.1 Performance evaluations

Accuracy (ACC), sensitivity (SN), specificity (SP), and area under the curve (AUC) (Wang et al., 2019) are often used to judge the quality of the proposed models. The accuracy rate represents the proportion of the sample that can be accurately predicted in the overall test sample. The larger the value of ACC, the higher the accuracy of the model’s prediction of the sample. However, it usually reflects only the overall situation of the sample. When evaluating models for imbalanced datasets, the ACC value can obscure some truth. In other words, the model’s prediction accuracy for samples from most categories may neutralize the low prediction accuracy of samples from a few categories. Therefore, we need to use other parameters for further analysis. Sensitivity represents the proportion of samples that are correctly predicted in positive samples, and specificity represents the proportion of samples that are correctly predicted in negative samples. The specificity and sensitivity reflect the actual predictions of the model for positive and negative samples. These two values will not change much due to the imbalance of the sample. Therefore, accuracy combined with sensitivity and specificity can objectively reflect the prediction of the model. The AUC value is based on the area enclosed by the receiver operating characteristic (ROC) curve and the coordinate axis. The ROC curve takes the sensitivity of the model as the ordinate and 1 minus the specificity as the abscissa. According to different classification thresholds, the relationship between sensitivity and specificity can be accurately analyzed. The AUC value is between 0 and 1. The larger the AUC value, the better the performance of the model.

### 4.2 Results

In this study, aiming to resolve the imbalance of children’s inguinal hernia text data, an integrated learning method based on the voting mechanism is used to reduce the impact of data imbalance. According to the characteristics of the sample data, blood routine and liver and kidney functions were used to establish different comprehensive classifying models. In this experiment, five-fold cross-validation (Mou et al., 2014) is used to further analyze the stability of the model. The ensemble of SVM and LR algorithms was compared with the current auxiliary diagnosis system. Table 4 lists the performance of different algorithms. M1 indicates that the model is constructed only from blood routine parameters, M2 indicates that the model is constructed only from liver and kidney function parameters, and M3 indicates that the model is constructed from all of the aforementioned parameters.

**TABLE 4:** Performance comparison of different models.

Feature set	Algorithm	ACC (%)	SN (%)	SP (%)	AUC
M1	RF	72.28	71.93	**79.30**	**0.72**
	SVM	**88.22**	**91.90**	15.67	0.53
	LR	74.28	74.44	71.27	0.71
M2	RF	86.15	85.93	**88.73**	**0.87**
	SVM	86.46	**87.01**	79.94	0.83
	LR	**86.51**	86.67	84.59	0.84
M3	RF	**82.47**	**79.35**	**98.14**	**0.89**
	SVM	77.87	77.50	79.72	0.76
	LR	78.59	76.04	91.42	0.83

The bold values mean they are the best performance of the same feature set in the metric labeled as the column heading.

**FIGURE 5:**
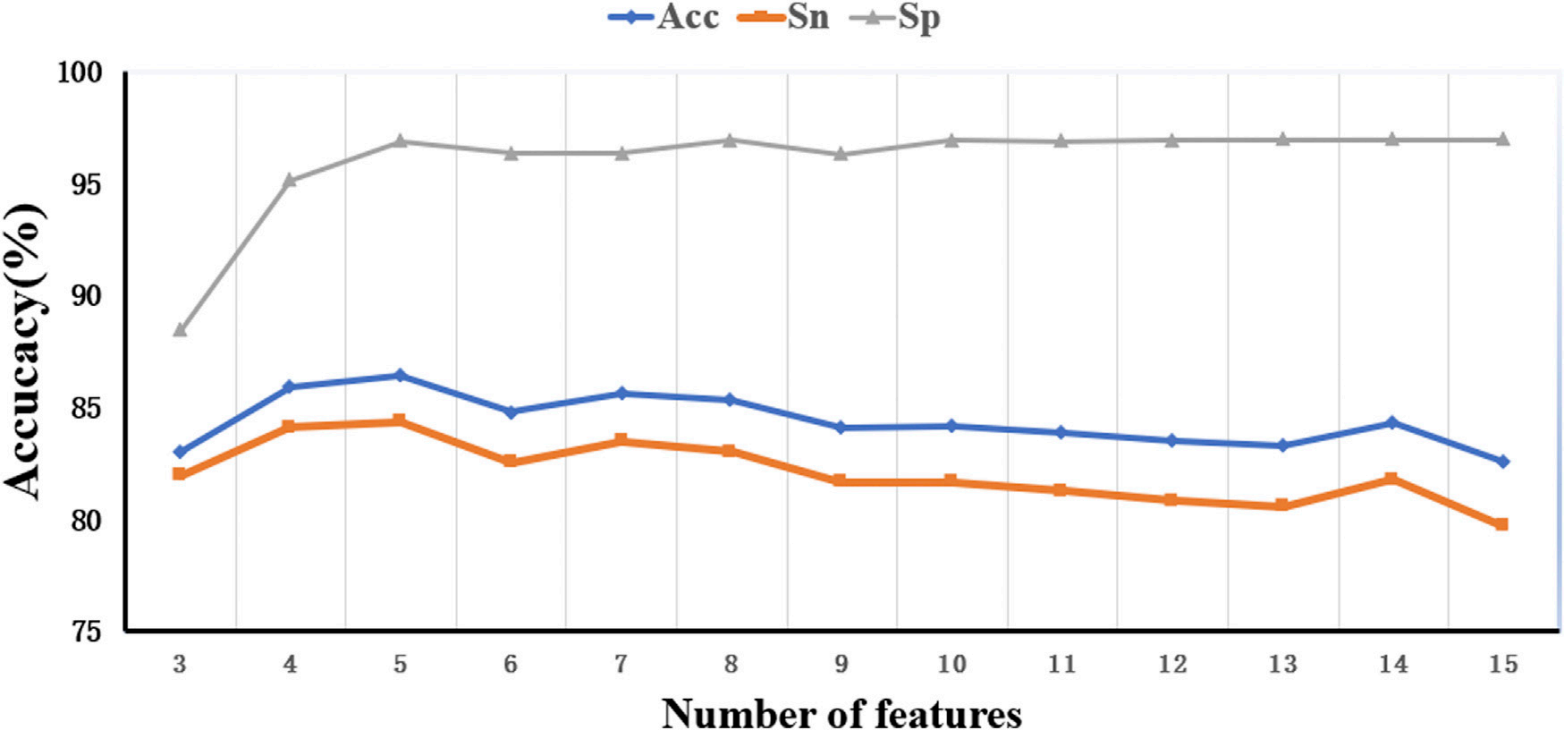
Trend of model performance under different quantitative characteristics.

**FIGURE 6:**
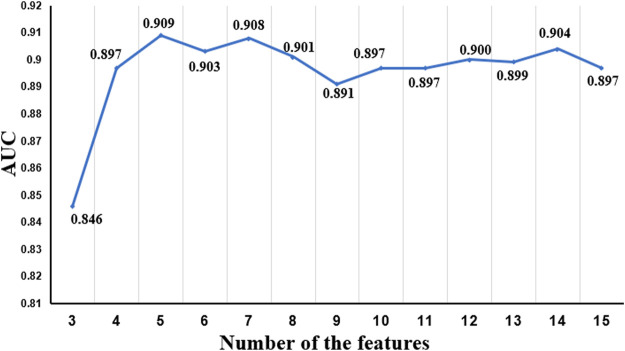
AUC values of different quantitative characteristics.

To optimize the performance of the RF algorithm trained with the M3 parameter, we further analyzed feature importance ranking, as shown in Table 3. Two items of research have been performed for the in-depth study. The first one is to screen out the intersection of the first 15 features of M3 and the first 10 features of M1 and M2 and then find the union of these two intersections. The union of these 11 features was denoted as FI and applied to train the enhanced model. The other one is to select the best feature combination from the aforementioned 15 features of M3. Since RF does not have a clear threshold for the feature importance, we continuously adjust the number of features in this experiment to achieve the goal of optimization, and the best combination feature set was denoted as FC_15. Figures 5, 6 show the performance of different feature sets, and the detailed results are listed in Table 5.

**TABLE 5:** Performance comparison of models based on feature selection.

Model	Algorithm	ACC (%)	SN (%)	SP (%)	AUC
M3	RF	82.47	79.35	98.14	0.89
FI	RF	81.75	78.73	96.91	0.89
FC_15	RF	**86.43**	**84.34**	**96.89**	**0.91**

The bold values mean they are the best performance of the same feature set in the metric labeled as the column heading.

### 4.3 Discussion

Table 4shows that the performances of the three models using the M1 parameters alone are not good, and the SVM algorithm even obtained a low specificity value (15.67%). The accuracies of models trained with M2 are all over 86%, and their sensitivity and specificity are prospective. As far as the overall performance is concerned, the performance of the RF algorithm under the M2 parameter is better than those of the models with M1 or M2 parameters. The values of ACC, SN, and SP of RF trained with M2 are 86.17%, 85.93%, and 88.73%, respectively. The reason why the performance of the models trained with M1 is inferior to that of the models with M2 may be that it is not effective enough for mining the information of children with intestinal necrosis from the blood routine features. The performance of the models with M3 is lower than that with M2, as shown in Table 4, and there are many interfering features which affect the involved models.

Figures 5, 6 show that when the feature number is 5, the performance of the model is better than the model under the M3 parameter. At this time, the five feature parameters are C-reactive protein, calcium, direct bilirubin, average hemoglobin, and the ratio of basophils. The AUC values of the model trained with M2 and M3 parameters are all larger than 0.87 (see Table 3), and the AUC value of the filtered characteristic FC_15 model can reach 0.91. Therefore, the author believes that the model constructed using medical text data can be used for a doctor’s auxiliary diagnosis. This work proved that the performance of a model can be further improved by selecting proper features with good priority.

To calculate the performance of the different models, the parameters used in this study can be summarized as follows: the random forest algorithm has n_estimators parameter of 200, criterion parameter of gini, min_samples_split parameter of 2, min_samples_leaf parameter of 1, and max_features parameter of “auto.” The support vector machine algorithm has C parameter of 1 and gamma of “scale.” The parameter in the logistic regression algorithm is 1e-4, the C parameter is 1, and the max_iter parameter is 100. All of these have been added in the MS.

## 5 Conclusion

The purpose of this study is to find the relationship between patients with intestinal necrosis and patients with inguinal hernia through blood routine and liver and kidney function test parameters, so as to provide auxiliary recommendations for children’s next treatment. Some constructive models were established on the heterogenetic feature sets and offer helpful answers to doctors’ diagnosis. Furthermore, our work highlighted many patient features that are predictive for making a diagnosis on the relevant diseases. For example, C-reactive protein parameters, troponin I, albumin, and total bilirubin are remarkably important for the issue. The vital sign parameters and image-type medical data would be helpful for the improved models.

Actually, routine blood tests and liver and kidney function tests are often overlooked by researchers because of their basic and common data. This study was conducted to explore the potential association of these basic clinical data with inguinal hernia disease and to construct a model to assist physicians in decision-making. Due to the imbalance of the clinical data and the sparsity of the features, the current study only attempts to use some conventional algorithms to train the samples for analysis. Therefore, this study is more of a trial, guided experiment. In the future, the authors aim to introduce more medical data and intelligent assistance.

However, there are a couple of limitations to this study. On the one hand, the obtained clinical data such as blood routine data and liver and kidney function test parameters are easily affected by the data collection process. If the clinical data parameters used in each study cannot be unified, some important parameters may be missed, which will increase some uncertain risks. On the other hand, due to the influence of research methods, clinical data are different from the characteristic values of some other sample data, and the normal value is usually given in a certain interval range. If one pays too much attention to the numerical weight of the parameters, the generalization ability of the model may be limited. Therefore, based on these limitations, in the future, the author will strictly use data standards and convert some parameters into codes by encoding, thereby weakening the individuality of parameter values.

## Data Availability

The raw data supporting the conclusion of this article will be made available by the authors, without undue reservation.
